# Dynamic sentiment spillovers among crude oil, gold, and Bitcoin markets: Evidence from time and frequency domain analyses

**DOI:** 10.1371/journal.pone.0242515

**Published:** 2020-12-03

**Authors:** Xianfang Su, Yong Li

**Affiliations:** School of Big Data Application and Economics, Guizhou University of Finance and Economics, Guiyang, P. R. China; The Bucharest University of Economic Studies, ROMANIA

## Abstract

This paper examines the sentiment spillovers among oil, gold, and Bitcoin markets by employing spillovers index methods in a time-frequency framework. We find that the total sentiment spillover among crude oil, gold and Bitcoin markets is time-varying and is greatly affected by major market events. The directional sentiment spillovers are also time-varying. On average, the Bitcoin market is the major transmitter of directional sentiment spillovers, whereas the crude oil and gold markets are the major receivers. In particular, the sentiment spillover effects are major created at high-frequency components, implying that the markets rapidly process the sentiment spillover effects and the shock is transmitted over the short-term. Moreover, we also find that the sentiment spillover effects differ significantly in term of intensity and direction when compared with return and volatility spillover effects. The present study has certain applications for investors and policymakers.

## Introduction

With advances in the study of behavioral finance, numerous scholars have realized that the prices of financial assets are not only based on intrinsic values and rational expectations, but are also driven by irrational factors such as investor sentiment [1–3]. Investor sentiment, defined as a belief about future cash flows and investment risks, reflects market investor’s emotional changes in speculative demand and has gained recognition as a new behavioral driving factor influencing financial asset price movements [4, 5]. Many existing studies have found that investor sentiment can serve as a price-discovery indicator to predict stock returns [6, 7]. In particular, numerous researchers examined the impacts of investor sentiment on cross-sectional stock returns and found that investor sentiment in one market will effect on the asset price in another market [8–12].

As a concept in the field of psychology, investor sentiment is a response to the market environment. Through conscious cognitive process, emotional responses, mimicry/feedback and other mechanisms, investor sentiment is transmitted from one individual to another [13], which is called the spillover effect of investor sentiment. In fact, investor sentiment spillover is the process by which investors acquire the sentiment of others, so as to realize the interaction and aggregation of opinions between different individuals. In recent years, sentiment spillover effect has attracted great attention because sentiment spillover among individual investors is one of the main sources of investor sentiment. In particular, investor sentiment and its spillover effect played a critical role in formation of systemic risk as the financial crisis unfolded [14]. However, although investor sentiment as an additional risk factor determining asset returns has been extensively investigated, studies on the spillover effects of investor sentiment are scant. To bridge this research gaps in sentiment spillover studies, the present article examines how investor sentiments in crude oil, gold and Bitcoin markets diffuse.

The purpose of this study is to examine the dynamic spillover effects of investor sentiments among crude oil, gold and Bitcoin markets. Moreover, we compare the different in direction and intensity of return spillover, volatility spillover and sentiment spillover in these three markets. There are two main reasons why we chose to focus on crude oil, gold and Bitcoin markets. Firstly, these three assets have been utilized as hedging assets by investors to offset their market risks and lock profits [15–17]. The conscious reasoning, analysis and imagination of investors holding these three assets lead to sentiment spillover effect [13]. For example, when the price of gold falls, investors consciously analyze the dependence of gold and Bitcoin and imagine that the price of Bitcoin will also fall, so the pessimism in the gold market will be transmitted to the Bitcoin market. Further, sentiment influences investors’ risk tolerance and subjective judgment, thereby affecting portfolio selection [18]. Thus, exploring the sentiment spillover effects among crude oil, gold and Bitcoin markets was deemed as enabling us to further gain insight into the hedging performances of safe haven assets.

Secondly, Bitcoin is the most popular cryptocurrency. As of September 1, 2019, Bitcoin has dominates the cryptocurrency market, with a market share of 67.43%. However, there are still mixed views on whether Bitcoin has an intrinsic value and whether its price is solely driven by factors such as perception and investor sentiment [19, 20]. As such, it was deemed to be interesting to investigate the investor sentiment feature in the Bitcoin market. Many studies have examined the impacts of investor sentiment on Bitcoin prices [21, 22]. However, to the best of our knowledge, there currently exists no literature on the sentiment spillover effects between the Bitcoin market and other markets.

In order to meet the research purpose of this article, we employed a time-frequency framework to capture the investor sentiment spillover effects across different financial markets. First, we used the spillover index method proposed by Diebold and Yilmaz [23] to explore the dynamic spillovers of sentiment among crude oil, gold, and Bitcoin markets. The spillover index method enables the measurement of the direction of spillovers based on forecast error variance decompositions from vector auto-regression models, and has been used in numerous subsequent empirical studies to examine the spillover effects in returns and volatility across individual assets [24–26]. Second, we employed a spectral representation of variance decompositions, as proposed by Barunik and Krehlik [27], to decompose the sentiment spillovers among the three markets into short-, medium-, and long-term components, and to analyze their dynamic behaviors. This helped us to further understand the dynamics of spillover effects among different financial markets at various frequency bunds [28]. At the same time, in order to further analyze the difference between sentiment spillover and return and volatility spillovers, we also used the time-frequency framework to examine the dynamics of return and volatility spillovers in the three markets, thus enabling us to understand the sentiment spillover effects among different financial markets in greater depth.

It is worth noting that we followed He [29] in constructing an investor sentiment index for the crude oil, gold, and Bitcoin markets in this study. In previous studies, the Google trend index and Twitter-based survey indicator have been a popular proxy for investor sentiment [30, 31]. However, the Google trend index is indifferent to whether searches are bullish or bearish, so it is not appropriate to use this index as a proxy for sentiment. In addition, the Twitter-based survey indicator can only be obtained through complicated web crawlers and natural language analysis. Therefore, the Twitter-based survey indicator will vary greatly depending on the software and algorithm used, and will lack robustness and comprehensiveness.

The index of investor sentiment provided by Baker and Wurgler [4], the VIX index constructed from implied volatilities of S&P 500 index options, and the consumer confidence index (CCI) complied by the Conference Board and the University of Michigan have also been widely used as sentiment proxy indexes in the stock market. However, these indexes are not specific ones that directly relate to investor sentiment in financial markets. The sentiment index we constructed assumes that all information will eventually be absorbed in closing prices, and measures the strength of both bullish and bearish investors through the probabilities of the highest and lowest prices eventually becoming closing prices. This sentiment index directly uses asset price differentials to measure investor reactions to all relevant news, and has been applied to sentiment analysis in financial markets [32, 33].

This study contributes to the existing literature on two fronts. First, it is the first study to use the time-frequency connectedness framework proposed by Barunik and Krehlik [27] to analyze the magnitude, direction and dynamics of investor sentiment spillover effects among crude oil, gold and Bitcoin markets. There is a realistic background for frequency decomposition of spillover dynamics. Since market participants operate on different investment horizons when they make investment decisions, the degree of dynamic spillover effects will differ at different frequencies. Thus, a frequency domain decomposition of dynamic spillovers enables us to understand the risk connectedness differences at different frequencies. It is crucial that investors with different investment horizons understand the frequency dynamics of various spillover effects.

Second, this study contributes to expand the investor sentiment literature. Despite intensive analysis, existing studies mainly focus on the impacts of investor sentiments on asset prices and ignore the spillover effect of investor sentiment. A variety of disciplines, including animal research, developmental psychology, clinical psychology, and social psychology, have proven that sentiment spillover is pervasive [13]. In reality, for individual investor, not only his own assessment of the fundamentals is important, but also his conjecture about the actions of other investors. Our results provide evidence for the long-standing intuition that investor sentiment will diffuse among different financial markets. In fact, measuring and analyzing the dynamic sentiment spillovers in oil, gold and Bitcoin markets, also makes it possible to examine the “fear of connectedness” expressed by market participants as they trade.

The main findings of the present study are as follows. First, the sentiment spillover effects among crude oil, gold and Bitcoin markets are time-varying and are greatly affected by major market events. Second, the direction of sentiment spillovers is also time-varying. On average, the Bitcoin market emerges as the major transmitter, whereas the crude oil and Bitcoin markets are the receivers. Before January 2019, the pairwise sentiments between the crude oil and gold market, and the crude oil and Bitcoin market were negative most of the time, whereas they present as almost positive after January 2019. Third, the major sentiment spillover effect is created at high-frequencies, indicating that the sentiment spillover effect is being transmitted over short-periods. Finally, the sentiment spillover was found to have a different magnitude and direction compared with return spillover and spillover.

The rest of this paper is structured as follows. Section 2 provides a concise literature review. Section 3 introduces the methodologies. Section 4 reports the data and descriptive statistics. Section 5 presents empirical results, and Section 6 concludes.

## Literature review

This paper draws on two main strands of literature. The first is the large body of literature examining the connections between crude oil, gold, and Bitcoin markets. A number of studies have directly examined the relationship between oil and gold markets. For example, Reboredo [15] used the copulas method to analyze the dependence structure between oil and gold prices. They found there to be a positive and significant average dependence between gold and oil, but tail independence between gold and oil markets. Their results indicate that gold can act as an effective safe haven against extreme oil price movements. Aguilera and Radetzki [34] compared the gold and oil markets to explain the surprisingly high correlation between oil and gold, and pointed out that development in the oil market influenced investment activity in gold, thus providing the most plausible explanation for the price synchronization of oil and gold prices. Their results show that oil prices rose first based on above-ground hurdles that restrained the capacity to produce, and gold prices then reacted as they were pushed up by rising safe-haven investments to store value. Khalfaoui [35] employed the DCC-MGARCH model and wavelet analysis method to examine the time-frequency performances of the oil-gold time varying nexus. Their results indicate a low nexus for the oil-gold pairing following the recent global financial crisis, and that gold and oil moved in reverse directions in the mid-run and long-run horizons during the crisis.

Since Bitcoin launched in 2009, it has shown great resilience during periods of turmoil, thereby often being proclaimed as digital gold. The relationships between Bitcoin, crude oil, and gold have drawn researchers’ interest. For example, Al-Yahyaee et al. [36] employed bivariate DCC-GARCH family models to examine the diversification and hedging properties of Bitcoin and gold assets for oil and S&P Goldman Sachs Commodity Index (GSCI) investors. They found that Bitcoin and gold provide diversification benefits for oil and the S&P GSCI, and confirmed the importance of Bitcoin and gold in oil and S&P GSCI portfolio management. At the same time, Das et al. [37] used a dummy variable GARCH and quantile regression model to examine the hedging and safe-haven properties of Bitcoin against crude oil implied volatility. Their results demonstrate that Bitcoin is not the superior asset over gold and US dollar to hedge oil related uncertainties.

In particular, Selmi et al. [38] used a quantile-on-quantile regression approach to assess the return spillovers among crude oil, gold and Bitcoin markets, finding that both Bitcoin and gold could serve a hedge role for oil price movements. They also indicated that the property seems to be sensitive to different market conditions of Bitcoin and gold, and to whether the oil price is in a downside, normal or upside regime. Further, Jin et al. [39] employed multifractal detrended cross-correlation analysis and multivariate GARCH models to investigate the multifractal cross correlations and volatility spillovers among crude oil, gold and Bitcoin markets. They found the dynamic correlations between gold and crude oil markets to be almost positive, and those between Bitcoin and gold, as well as those between Bitcoin and oil markets, to be nearly negative throughout the sample periods. Moreover, their results show that significant volatility spillovers can be detected among the crude oil, gold and Bitcoin markets, and that the latter is mainly an information receiver, while the gold market is the major information transmitter in the system of hedging assets. However, how investor sentiments spillover across the three markets is still an open question.

Secondly, this current paper also relates to the existing body of literature investigating investor sentiment in financial markets. Baker and Wurgler [4, 5] constructed an investor sentiment index and found that investor sentiment can be used to predict stock returns. Further, Stambaugh, Yu and Yuan [40] examined the impact of investor sentiment on a broad set of anomalies in cross-sectional stock returns. They confirmed that investor sentiment exert an asymmetric effect on stock returns and proposed that high sentiment is more likely to produce overpricing than low sentiment. In particular, Pant et al. [21] used neural networks to analyze investor sentiment, with reference to predictive power of Twitter regarding the volatile price of Bitcoin. They found that the overall price prediction accuracy of the RNN model reached 77.62%. Karalevicius [22] indicated that media sentiment is a good short-term predictor and driver of Bitcoin price movements. However, they also emphasized that this would not allow someone to make exceptional profits, and that the speculative nature of Bitcoin should not be overstated. Vidal-Tomás et al. [41] analyzed the existence of herding in the cryptocurrency market. They observed herding during down markets. They also found the smallest digital currencies to be herding with the largest ones. However, the study does not examine the sentiment spillover effects between cryptocurrency and other financial assets.

Numerous researches investigated the impact of investor sentiment in one stock market on the returns of stock in other markets, and they considered this process as sentiment contagion. For example, Baker, Wurgler and Yuan [8] decomposed this total sentiment into one global sentiment and six local sentiments. Using the regression analysis, they found that cross-market sentiment contagion is part of the cause of global sentiment. Moreover, their result showed that both global and local sentiments have impacts on cross-sectional stock returns. Lee and Tucker [9] employed the stock market volatility to replace investor sentiment and adopted a bivariate GARCH model to examine the global market sentiment propagates. They found that a global contagion of investor sentiment occurred from the US market to other main stock markets during the course of the US subprime crisis. Hudson and Green [10] used principle component method to construct investor sentiment for UK investors and US investors. Employing the Granger causality test, they found that sentiment tends to be a more important determinant of returns in turbulent periods than tranquil periods. Moreover, they found that US investor sentiment is highly significant in explaining the UK stock returns. However, it is worth noting that the term ‘*sentiment contagion’* mentioned in the above literature is different from the present article.

Our study is closely related to Tsai [11]. This paper also employs the spillover index framework proposed by Diebold and Yilmaz [23] to examine the sentiment spillover effect among foreign investors, trust investors, and dealers in Taiwan stock market. Tsai [11] used the buy-sell imbalance (BSI) to construct investor sentiment index and found that the sentiments of trust investors and dealers have impact on foreign investors, and that the sentiment of dealers has impact on trust investors. Compared with his research on the sentiment spillover effect between different types of investors in the same market, our paper studies the spillover effect of investor sentiment among different asset markets. Moreover, in addition to examining the time-domain dynamic of investor sentiment spillover, we also investigate its frequency-domain characteristics of different frequency ranges.

## Methodology

### Measuring time domain spillover effects

Let us consider a standard N-variable VAR(p) framework, $X_{t}=\sum_{j=1}^{p}\Phi_{j}X_{t-j}+\varepsilon_{t}$, where *ε*_*t*_~*i*.*i*.*d*.(0,Σ) is a vector of error, and Σ is the variance of error terms. The VAR model can be rewritten into a moving average representation as *X*_*t*_ = Ψ(*L*)*ε*_*t*_, where Ψ(*L*) is an *n*×*n* infinite lag polynomial matrix of coefficients. Following Diebold and Yilmaz [23], we decomposed the shocks using a generalized vector autoregressive framework. The main benefit of the generalized VAR framework is that the forecast-error variance decompositions are invariant to the ordering of the variables in the VAR framework. The H-step-ahead generalized forecast error variance decompositions can be defined as follows:

$$\theta_{jk}(H)=\frac{\sigma_{kk}^{-1}\sum_{h=0}^{H-1}{\left({\left(\Psi_{h}\Sigma \right)}_{jk}\right)}^{2}}{\sum_{h=0}^{H-1}{\left(\Psi_{h}\Sigma {\Psi^{\prime }}_{h}\right)}_{jj}},$$ (1)

where *σ*_*kk*_ is the standard deviation of the error term for the *k*th equation, and Ψ_*h*_ is an *n*×*n* matrix of coefficients corresponding to lag *h*. *θ*_*jk*_(*H*) denotes the contribution of the *k*th asset of the system to the variance of the forecast error of element, *j*. In order to ensure that the sum of the elements of each contribution of the variance decomposition is equal to 1, each forecast error variance decomposition is normalized by the row sum as:

$${\tilde{\theta }}_{jk}(H)=\frac{\theta_{jk}(H)}{\sum_{k=1}^{N}\theta_{jk}(H)}.$$ (2)

Using the normalized forecast error variance decomposition, ${\tilde{\theta }}_{jk}(H)$, we can define the total spillover index (TSI), directional spillover index (DSI), and net spillover index (NSI). The total spillover index (TSI) is the sum of cross-variance, denoting the fractions of the H-step-ahead error variances in forecasting *X*_*j*_ that are due to shocks to *X*_*k*_. The TSI is defined as follows:

$$TSI(H)=\frac{\sum_{j,k=1,j\neq k}^{N}{\tilde{\theta }}_{jk}(H)}{\sum_{j,k=1}^{N}{\tilde{\theta }}_{jk}(H)}\times 100.$$ (3)

The directional spillover index (DSI) captures the shocks received by vector *j* from all other vectors. The directional spillover index from vector *j* to all other vectors can be defined in a similar manner. The first directional spillover index (“From” directional spillover index) is measured as follows:

$$DSI_{j•}(H)=\frac{\sum_{k=1,k\neq j}^{N}{\tilde{\theta }}_{jk}(H)}{\sum_{k=1}^{N}{\tilde{\theta }}_{jk}(H)}\times 100.$$ (4)

The second directional spillover index (“To” directional spillover index) is similarly measured as follows:

$$DSI_{•k}(H)=\frac{\sum_{j=1,j\neq k}^{N}{\tilde{\theta }}_{jk}(H)}{\sum_{j=1}^{N}{\tilde{\theta }}_{jk}(H)}\times 100.$$ (5)

The net spillover index (NSI) from vector *j* to all other vectors is simply the difference between the “To” directional spillover index and the “From” directional spillover index, i.e.:

$$NSI_{j}(H)=DSI_{•j}(H)-DSI_{j•}(H).$$ (6)

In order to capture the time-varying nature of the spillover index, we applied a rolling-window methodology using the same measures described above to measure the dynamic spillover index.

### Measuring frequency domain spillover effects

To measure frequency connectedness, we employed the spectral representation of variance decompositions proposed by Barunik and Krehlik [27]. For the frequency band *D* = (*a*,*b*):*a*,*b*∈(−*π*,*π*),*a*<*b*, we amied to obtain the total spillover index (TSI), directional spillover index (DSI), and net spillover index (NSI) on the frequency band *D*. As mentioned above, once the infinite lag polynomial matrix of coefficients, Ψ(*L*), had been obtained, this made it possible to define the generalized forecast error variance decompositions on frequency band *D*, as follows:

$$\theta_{jk}(D)=\frac{1}{2\pi }\int_{D}\Gamma_{j}(\omega ){\left(f(\omega )\right)}_{jk}d\omega ,$$ (7)

where,

$${\left(f(\omega )\right)}_{jk}=\frac{\sigma_{kk}^{-1}{\left|{\left(\Psi (e^{-iw})\Sigma \right)}_{jk}\right|}^{2}}{{\left(\Psi (e^{-iw})\Sigma \Psi^{\prime }(e^{+iw})\right)}_{jj}},$$ (8)

$$\Gamma_{j}(\omega )=\frac{{\left(\Psi (e^{-iw})\Sigma \Psi^{\prime }(e^{+iw})\right)}_{jj}}{\frac{1}{2\pi }\int_{-\pi }^{\pi }{\left(\Psi (e^{-i\lambda })\Sigma \psi^{\prime }(e^{+i\lambda })\right)}_{jj}d\lambda },$$ (9)

In more detail, $\Psi (e^{-iw})=\sum_{h}e^{-iwh}\psi_{h}$ is the spectral representation of coefficient matrix Ψ_*h*_. Therefore, the (*f*(*ω*))_*jk*_ denotes the portion of the spectrum of the *j*th asset at frequency *ω* that are due to shocks in the *k*th asset. Γ_*j*_(*ω*) is the weighting function of the frequency share of variance for the *j*th asset, which denotes the power of the *j*th asset at a given frequency.

Further, the generalized forecast error variance decompositions on the frequency band *D* can be scaled as:

$${\tilde{\theta }}_{jk}(D)=\frac{\theta_{jk}(D)}{\sum_{k=1}^{N}\theta_{jk}(\infty )},$$ (10)

where $\theta_{jk}(\infty )=\sum_{d_{s}\in D}\theta_{jk}(d_{s})$. Employing the generalized forecast error variance decompositions on the frequency band *D*, the total spillover index (TSI), directional spillover index (DSI), and net spillover index (NSI) on the frequency band *D* can be defined as:

$$TSI(D)=\frac{\sum_{j,k=1,j\neq k}^{N}{\tilde{\theta }}_{jk}(D)}{\sum_{j,k=1}^{N}{\tilde{\theta }}_{jk}(D)}\times 100,$$ (11)

$$DSI_{j•}(D)=\frac{\sum_{k=1,k\neq j}^{N}{\tilde{\theta }}_{jk}(D)}{\sum_{k=1}^{N}{\tilde{\theta }}_{jk}(D)}\times 100,$$ (12)

$$DSI_{•k}(D)=\frac{\sum_{j=1,j\neq k}^{N}{\tilde{\theta }}_{jk}(D)}{\sum_{j=1}^{N}{\tilde{\theta }}_{jk}(D)}\times 100,$$ (13)

$$NSI_{j}(D)=DSI_{•j}(D)-DSI_{j•}(D).$$ (14)

It is here worth noting that the spillover index is equivalent to the *within connectedness* of Barunik and Krehlik [27], by which we could decompose the original connectedness into distinct frequency parts that, in sum, yielded the original connectedness measure.

### Data and descriptive statistics

**Fig 1:**
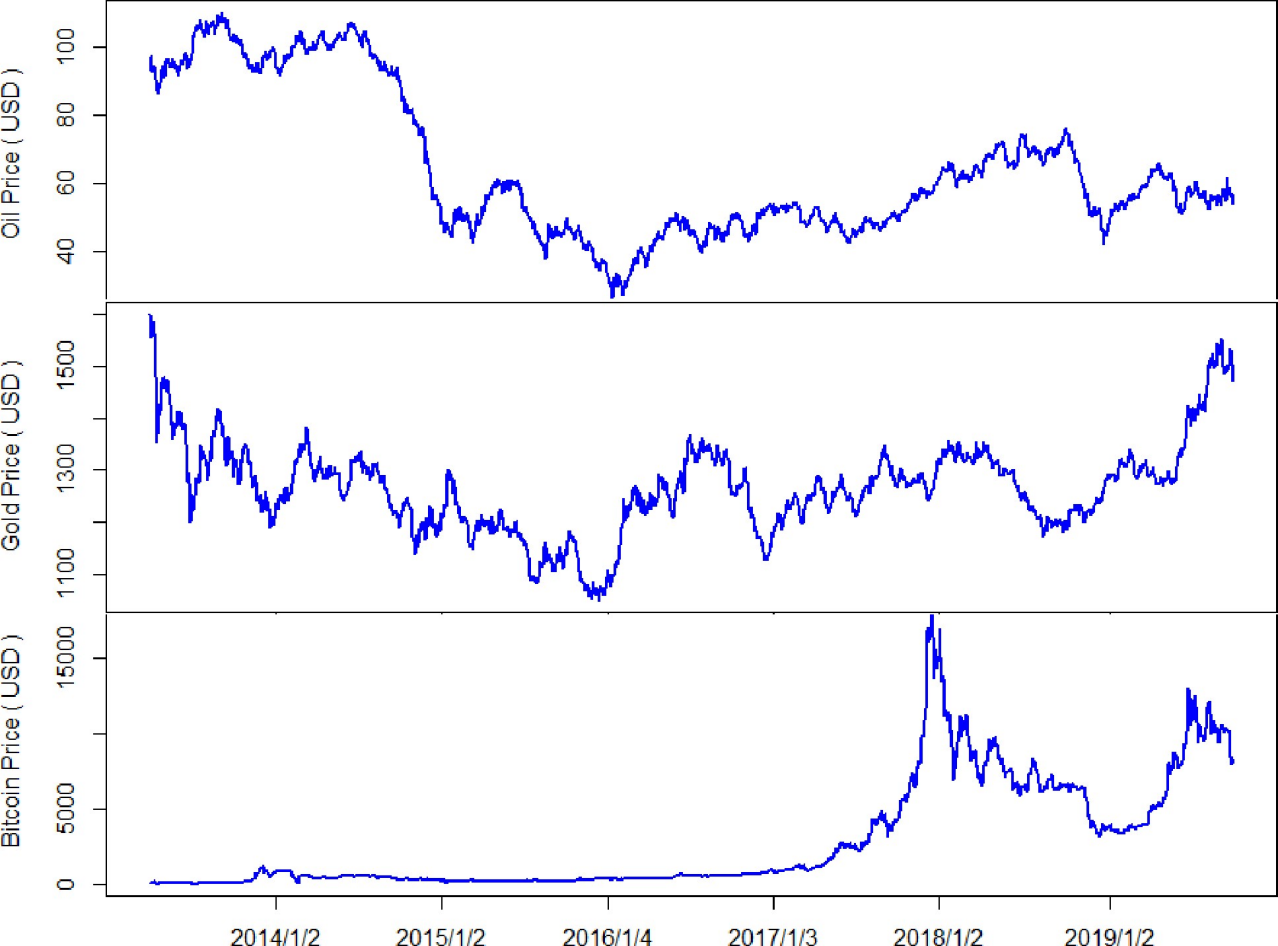
Price dynamics of oil, gold, and Bitcoin from April 2013 to September 2019.

This study employed the daily trading data of crude oil, gold, and Bitcoin. The spot prices of gold are quoted as US dollars per troy ounce, whereby the data source is the London PM fix (www.kitco.com). The West Texas Intermediate (WTI) crude oil prices (US dollars per barrel) are available from the US Energy Information Administration (EIA) website (www.eia.gov). The Bitcoin trading activity data were collected from Coinmarketcap.com. For each price series, the closing price, opening price, highest price, and lowest price were available. We studied the period spanning April 1, 2013, to September 30, 2019. This sample period was chosen because the market value of Bitcoin exceeded 1 billion USD on March 30, 2013, and the Bitcoin price reached 100 USD on April 1, 2013. To maintain a uniform measurement interval across markets, we excluded days when any of the three markets were closed. This procedure yielded 1,649 observations for each market. Fig 1 shows the daily closing prices of crude oil, gold, and Bitcoin over the study period. We have observed the price comovement between crude oil and gold markets. In particular, after the price of Bitcoin reached $2883 on July 31, 2017, its trend was similar to those of crude oil and gold.

The returns were constructed using *r*_*t*_ = 100×ln(*p*_*t*_/*p*_*t*−1_), where *p*_*t*_ represents daily returns in period *t*. Following Alizadeh et al. [42], we took the difference between the natural logarithms of the daily high, low, opening, and closing prices to construct the following daily range-based volatility estimation:

$${\tilde{\sigma }}_{t}^{2}=0.511{\left(h-l\right)}^{2}-0.019[(c-o)(h+l-2o)-2(h-o)(l-o)]-0.383{\left(c-o\right)}^{2},$$ (15)

where *h*,*l*,*o* and *c* denote, respectively, the natural logarithms of daily high, low, opening, and closing prices in market *i* on day *t*.

To measure investor sentiment, the following binomial probability distribution model was described:

$$P_{t}\times H_{t}+(1-P_{t})\times L_{t}=C_{t},$$ (16)

where *P*_*t*_ refers to the possibility of the highest price (*H*_*t*_) being the closing price (*C*_*t*_), and takes a value of zero to unity; and (1-*P*_*t*_) represents the possibility of the lowest price (*L*_*t*_) being the closing price. When *P*_*t*_>0.5, the overall investor sentiment is optimistic; if *P*_*t*_ = 0.5, the overall investor sentiment is considered neutral; while *P*_*t*_<0.5 indicates that the overall investor sentiment is pessimistic. Therefore, the investor sentiment index can be further expressed as:

$$IS_{t}=P_{t}-0.5,$$ (17)

where a positive *IS* represents an optimistic sentiment toward the closing price; while a negative *IS* indicates a pessimistic sentiment toward the closing price.

Table 1 presents the descriptive statistics of return, volatility, and sentiment in crude oil, gold, and Bitcoin markets. The Shapiro-Wilk statistics highlight that none of the return, volatility and sentiment series are normally distributed. Further, the augmented Dickey-Fuller (ADF) test and Phillips and Perron (PP) test were conducted to test the stationarity for the return, volatility, and sentiment in crude oil, gold, and Bitcoin markets. The subsequent results clearly show that all the series are stationary at the 1% significance level.

**Table 1:** Descriptive Statistics of return, volatility, and sentiment in crude oil, gold, and Bitcoin markets.

	Min.	Max.	Mean	Std. Dev.	Skewness	Kurtosis	Shapiro-Wilk test	PP test	ADF test
Oil-Return	-0.1010	0.1243	-0.0002	0.0207	0.1964	5.9099	0.9690***	-1735.8***	-29.0732***
Oil-Volatility	0.0000	0.0800	0.0178	0.0101	1.55520	6.4192	0.7827***	-838.64***	-13.3139***
Oil-Sentiment	-0.5000	0.5000	0.0119	0.3093	-0.0321	1.6533	0.9358***	-1794.9***	-28.3109***
Gold-Return	-0.0849	0.0480	-0.0001	0.0091	-0.5572	10.2674	0.9447***	-1770.4***	-29.1716***
Gold-Volatility	0.0000	0.0600	0.0086	0.0052	1.1517	13.8588	0.6126***	-1781.4***	-22.8242***
Gold-Sentiment	-0.5000	0.5000	-0.0066	0.2836	0.0583	1.7114	0.9472***	-1817.4***	-28.5992***
Bitcoin-Return	-0.5721	0.4867	0.0023	0.0541	-0.4083	22.1296	0.8157***	-1707.0***	-26.8795***
Bitcoin-Volatility	0.0000	0.6700	0.0398	0.0473	5.5826	52.4519	0.5431***	-648.01***	-13.6471***
Bitcoin-Sentiment	-0.5000	0.5000	0.0475	0.2631	-0.1767	1.9203	0.9637***	-1872.5***	-26.1404***

Notes

*** represents significance at 1% level.

## Empirical results

Following the methodology described in Section 3, we used the Akaike Information Criterion (AIC) criteria to automatically choose the lag length of the three variable VAR models. A 100-period ahead forecasting horizon was used to construct the volatility forecast errors. At the same time, we used 200-day rolling windows to estimate the dynamic spillover effects. In order to check the validity of the results, we also used 150-day and 250-day rolling windows to estimate the dynamic return, volatility, and sentiment spillover index, by which we obtained the same dynamic behavior characteristics as when using a 200-day rolling window. In the frequency decomposition of spillover index, the frequency bands 3.14–0.63, 0.63–0.16 and 0.16–0.00 correspond to the short-term (1 day to 5 days), medium-term (5 days to 20 days), and long-term (20 days to 200 days) spillover components, respectively. Following this process, we examined the total and net spillover dynamics of return spillover, volatility spillover, and sentiment spillover in the time-frequency domain.

### Sentiment spillovers among crude oil, gold, and Bitcoin markets

**Fig 2:**
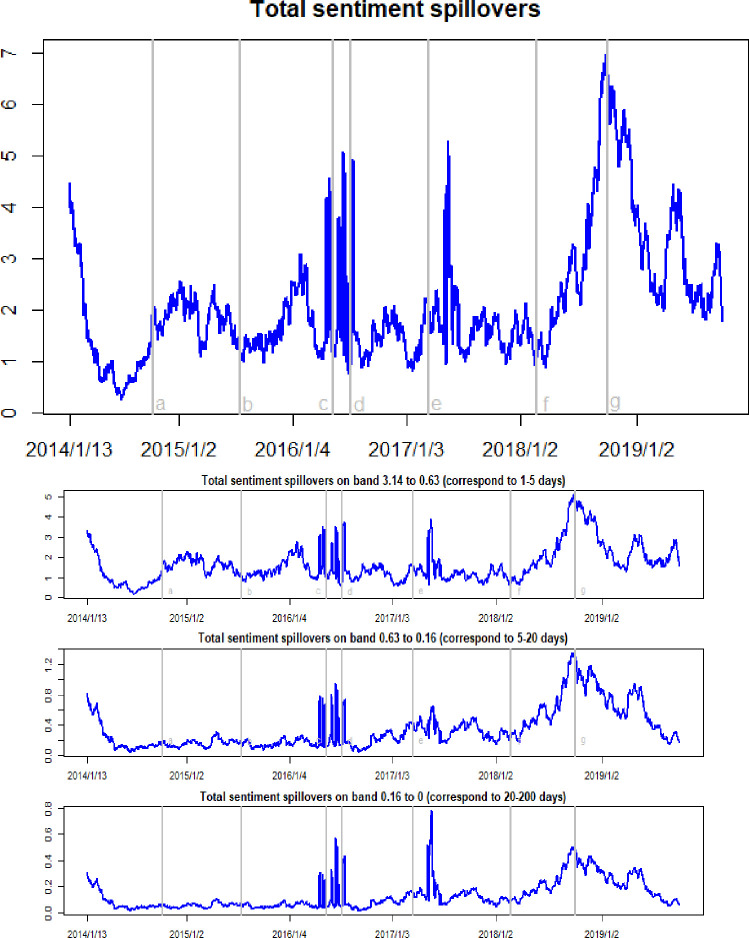
Time-frequency dynamics of total sentiment spillovers. a, b, c, d, e, f, g respectively represent the seven events: the WTI oil price collapsed in October 2014, the black swan event in gold market occurred in July 2015, the sharp rise of bitcoin prices in May 2016, the Brexit event in July 2016, the expectations of a Federal Reserve rate hike in March 2017, the price of Bitcoin and gold fluctuating fall at the beginning of 2018, and the WTI oil price declines in August 2018.

Fig 2 reports the time-frequency dynamics of total sentiment spillovers among crude oil, gold, and Bitcoin markets. The total sentiment spillover measures the percentage of sentiment variation caused by external markets to all sentiment variation in the entire system. Here, it can be seen that the total sentiment spillover ranges between 0.25 and 6.98, with a substantial variation over the sample period. This means that from 0.25 percent to 6.98 percent of the variation in investor sentiment in the entire market is due to investor sentiment spillovers between different markets. Such a variation is to be expected because the prices of these three assets changed dramatically during the study period, in which investor sentiment shocks were transmitted across these markets with different intensities. In fact, the investor sentiment index used in the present paper directly uses asset price differentials to quantify investor reactions to all relevant information. It can play a significant role in explaining variations in the crude oil, gold, and Bitcoin markets. The total sentiment spillovers peaked in the third quarter of 2018, when crude oil prices, gold prices, and Bitcoin prices all experienced a sharp fall. The decline in market prices caused the spread of investor pessimism and, ultimately, led to strong sentiment spillover effects in these markets. This conclusion can also be confirmed from the other period when the total sentiment spillovers sharply increased. Following the WTI oil price collapse in October 2014, the subsequent Brexit event in July 2016, and the Federal Reserve rate hike in March 2017, the total sentiment spillover effects appeared in discrete upward jumps.

The right column in Fig 2 shows the frequency domain decompositions of the total sentiment spillover dynamics. The low-, medium-, and high-frequency components of total sentiment spillovers show a similar variation mode, with different magnitudes over the sample period. The high-frequency component shows the maximum magnitude, with ranges between 0.17 and 5.14. As shown in Table 2, the average values of the high-, medium- and low-frequency are 1.65, 0.34 and 0.13, respectively. These data highlight that the total sentiment spillovers among the three markets is higher in the short-run rather than in the long-run, revealing that the shocks creating large connectivity in the total sentiment spillover impact the markets in the short term. In fact, investor sentiment changes rapidly as asset prices vary. When asset prices rise, investors are optimistic about the markets and, when asset prices fall, they become pessimism. Therefore, it can be said that the sentiment shock to one asset in the markets mainly affects short-term cyclical behavior.

**Table 2:** The statistical results of time-frequency dynamics of sentiment spillovers in crude oil, gold, and Bitcoin markets.

	Time domain spillovers			High frequency spillovers			Medium frequency spillovers			Low frequency spillovers		
	Min.	Max.	Mean	Min.	Max.	Mean	Min.	Max.	Mean	Min.	Max.	Mean
Total	0.25	6.98	2.13	0.17	5.14	1.65	0.03	1.35	0.34	0.01	0.78	0.13
Net-oil	-1.30	2.43	-0.02	-1.28	1.58	0.02	-0.30	0.50	-0.006	-0.11	0.57	0.001
Net-gold	-2.59	1.17	-0.03	-2.38	1.15	-0.04	-0.41	0.18	-0.007	-0.19	0.07	-0.003
Net-Bitcoin	-0.93	1.55	0.05	-0.97	1.60	0.02	-0.40	0.42	0.01	-0.50	0.18	0.002

**Fig 3:**
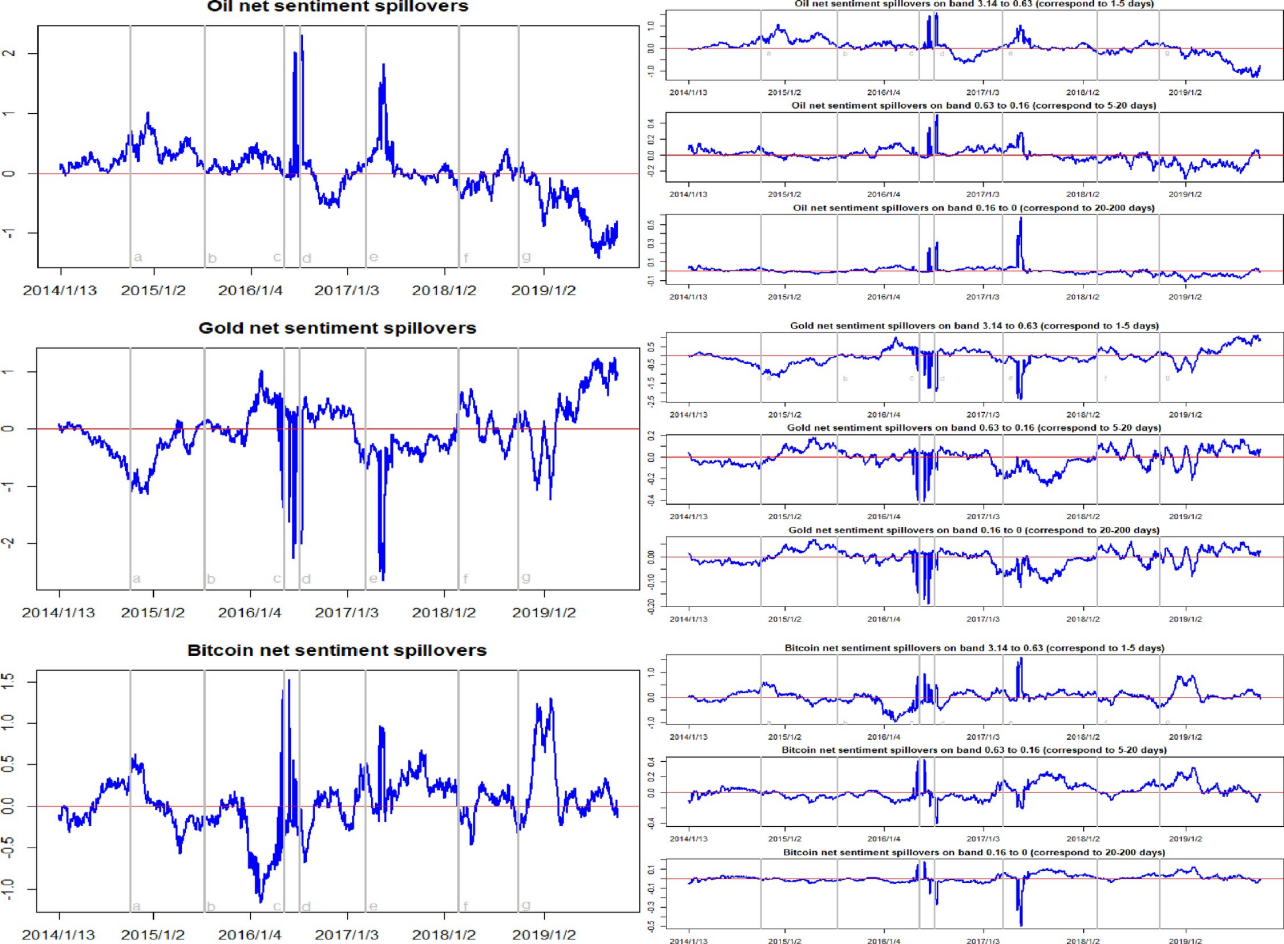
Time-frequency dynamics of net sentiment spillovers for oil, gold, and Bitcoin. a, b, c, d, e, f, g respectively represent the seven events: the WTI oil price collapsed in October 2014, the black swan event in gold market occurred in July 2015, the sharp rise of bitcoin prices in May 2016, the Brexit event in July 2016, the expectations of a Federal Reserve rate hike in March 2017, the price of Bitcoin and gold fluctuating fall at the beginning of 2018, and the WTI oil price declines in August 2018.

The time-frequency dynamics of net sentiment spillovers are presented in Fig 3. Focusing on the net sentiment spillover dynamics in the left column in Fig 3, it can be seen that the Brexit event in July 2016 and the Federal Reserve rate hike event in March 2017 exerted a great impact on the investor sentiment spillover effects. With the occurrence of these two events, the crude oil and the Bitcoin markets acted as the transmitters of net sentiment spillovers, with the gold market being the receiver of the net sentiment spillovers. Moreover, looking at the entire sample period, the net sentiment spillovers of the Bitcoin market can be seen to be positive most of the time. This indicates that the Bitcoin market is the major market sentiment transmitter out of these three hedging assets market.

This can be confirmed more concretely by considering the data presented in Table 2. Here the average net sentiment spillover of the Bitcoin market is 0.05, while the average net sentiment spillovers of the crude oil and gold markets are -0.02 and -0.03. In other words, on average, investors are more concerned about the variations in the Bitcoin market. Therefore, variations in sentiment in the Bitcoin market will transmit to the crude oil and gold markets. A plausible explanation behind this result is that Bitcoin is a newly introduced financial asset with no fundamental value. Its investors are driven only by the expected profits of acquiring Bitcoins and selling them later. The Bitcoin market is thus dominated by short-term investors and speculators. In addition, if we consider the frequency domain decomposition dynamics shown in the right column in Fig 3, we find that the shocks caused by sentiment spillovers are not persistent, and are transmitted for shorter periods.

**Fig 4:**
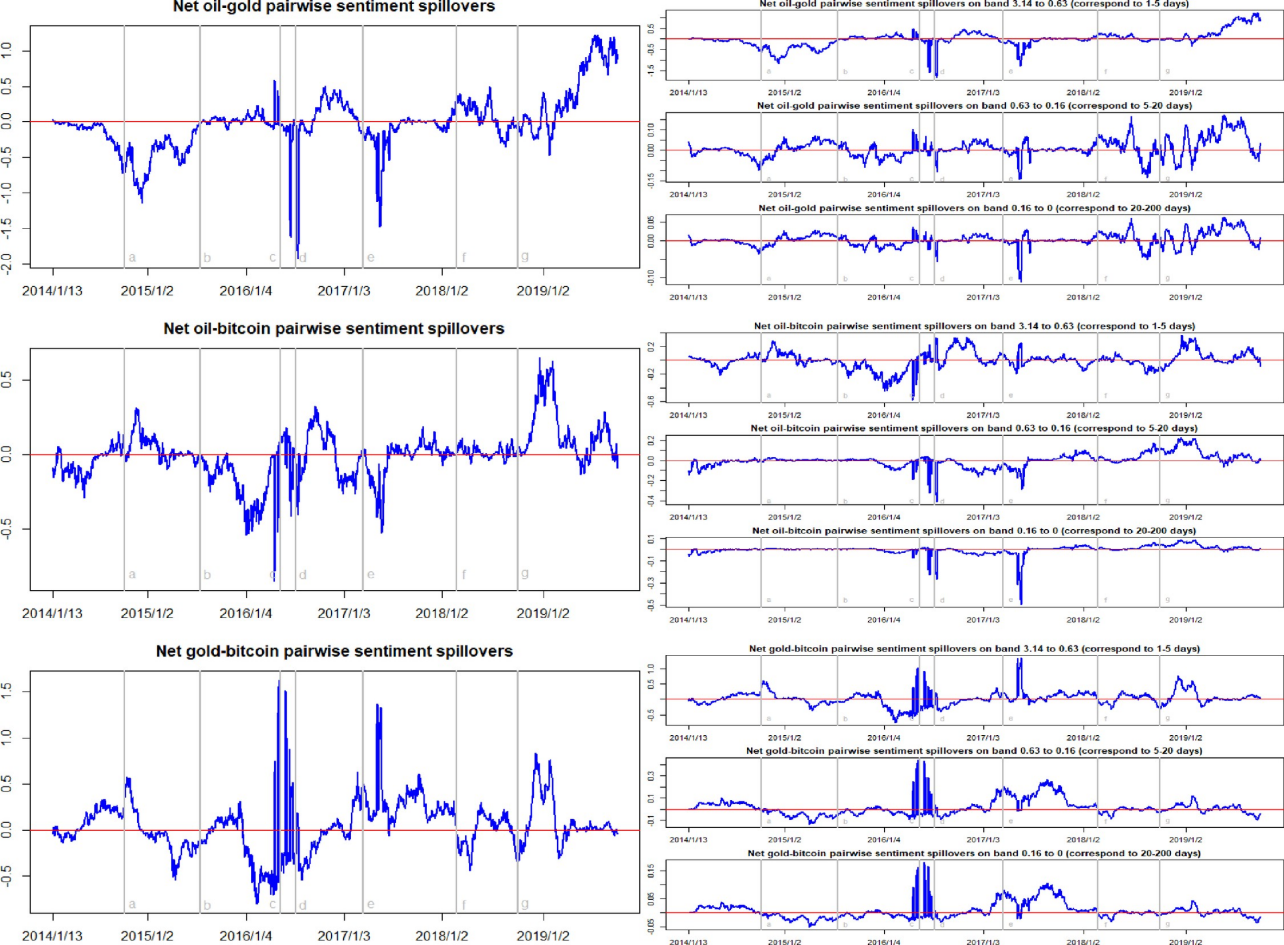
Time-frequency dynamics of net pairwise sentiment spillovers among oil, gold, and Bitcoin. a, b, c, d, e, f, g respectively represent the seven events: the WTI oil price collapsed in October 2014, the black swan event in gold market occurred in July 2015, the sharp rise of bitcoin prices in May 2016, the Brexit event in July 2016, the expectations of a Federal Reserve rate hike in March 2017, the price of Bitcoin and gold fluctuating fall at the beginning of 2018, and the WTI oil price declines in August 2018.

Fig 4 reports the net pairwise sentiment spillovers among oil, gold and Bitcoin markets. The net pairwise sentiment spillover index can point out which of the two markets is the sentiment transmitter and which is the receiver. For example, the ‘net oil-gold pairwise sentiment spillover’ is equal to the sentiment spillover from the crude oil market to the gold market minus the sentiment spillover from the gold market to the crude oil market. If the value of net oil-gold pairwise sentiment spillover is positive, it means that the crude oil market is the transmitter of sentiment, and conversely, it means that the crude oil market is receiver. The most obvious feature here is that the net pairwise sentiment spillovers between the assets is highly volatile, and shifts between positive and negative. Before January 2019, the net pairwise directional sentiment spillovers between the oil and gold markets, and between the oil and Bitcoin markets held negative values most of the time. This implies that the oil market is the receiver of sentiment shocks created by the gold and Bitcoin markets. However, after January 2019, the net pairwise sentiment spillovers are positive between oil and gold markets, oil and Bitcoin markets. The results of these pairwise sentiment spillovers further confirm the conclusion relating to net sentiment spillovers. Based on the results of the frequency decomposition, we find that the pairwise sentiment spillovers at high frequency yield similar results to those of the overall pairwise sentiment spillovers.

The frequency domain decomposition dynamics of the net pairwise sentiment spillovers are shown on the right column in Fig 4. For the net oil-gold sentiment spillover the magnitude of high frequency component is significantly higher than the medium and low frequency components. This indicates that in the crude oil market and the gold market, the sentiment spillover effect between short-term investors is more significant than that of medium- and long-term investors. The reason behind this may be that the short-term investors have a stronger subjective willingness to speculate and analyze the variations in market information. Similar characteristics appear in the net gold-Bitcoin pairwise sentiment spillover, in which the high-frequency components dominate the spillover index. However, for the net oil-Bitcoin pairwise sentiment spillover, the amplitudes of high, medium, and low-frequency components are not significantly different.

### Return and volatility spillovers among crude oil, gold, and Bitcoin markets

**Fig 5:**
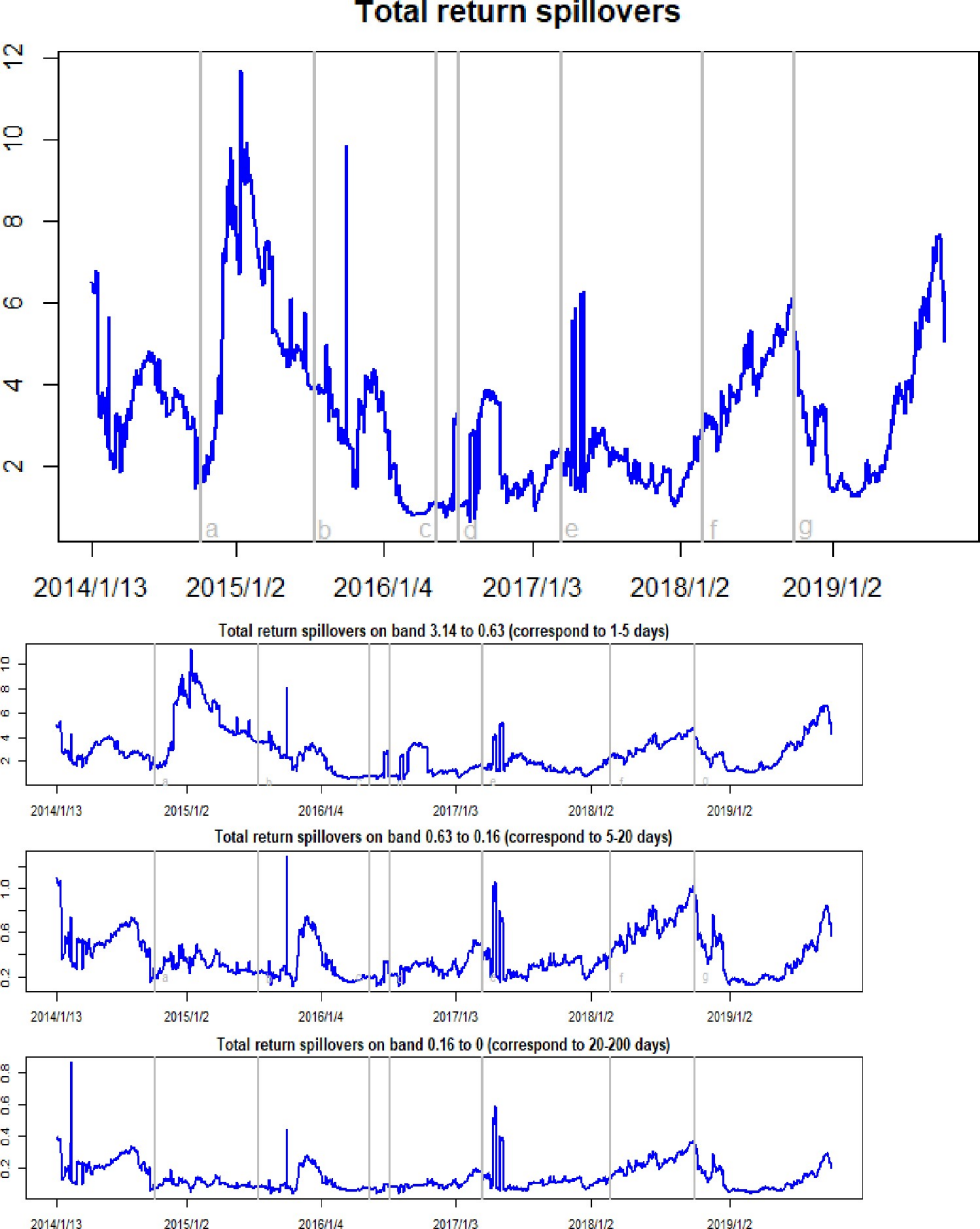
Time-frequency dynamics of total returns spillovers. a, b, c, d, e, f, g respectively represent the seven events: the WTI oil price collapsed in October 2014, the black swan event in gold market occurred in July 2015, the sharp rise of bitcoin prices in May 2016, the Brexit event in July 2016, the expectations of a Federal Reserve rate hike in March 2017, the price of Bitcoin and gold fluctuating fall at the beginning of 2018, and the WTI oil price declines in August 2018.

**Fig 6:**
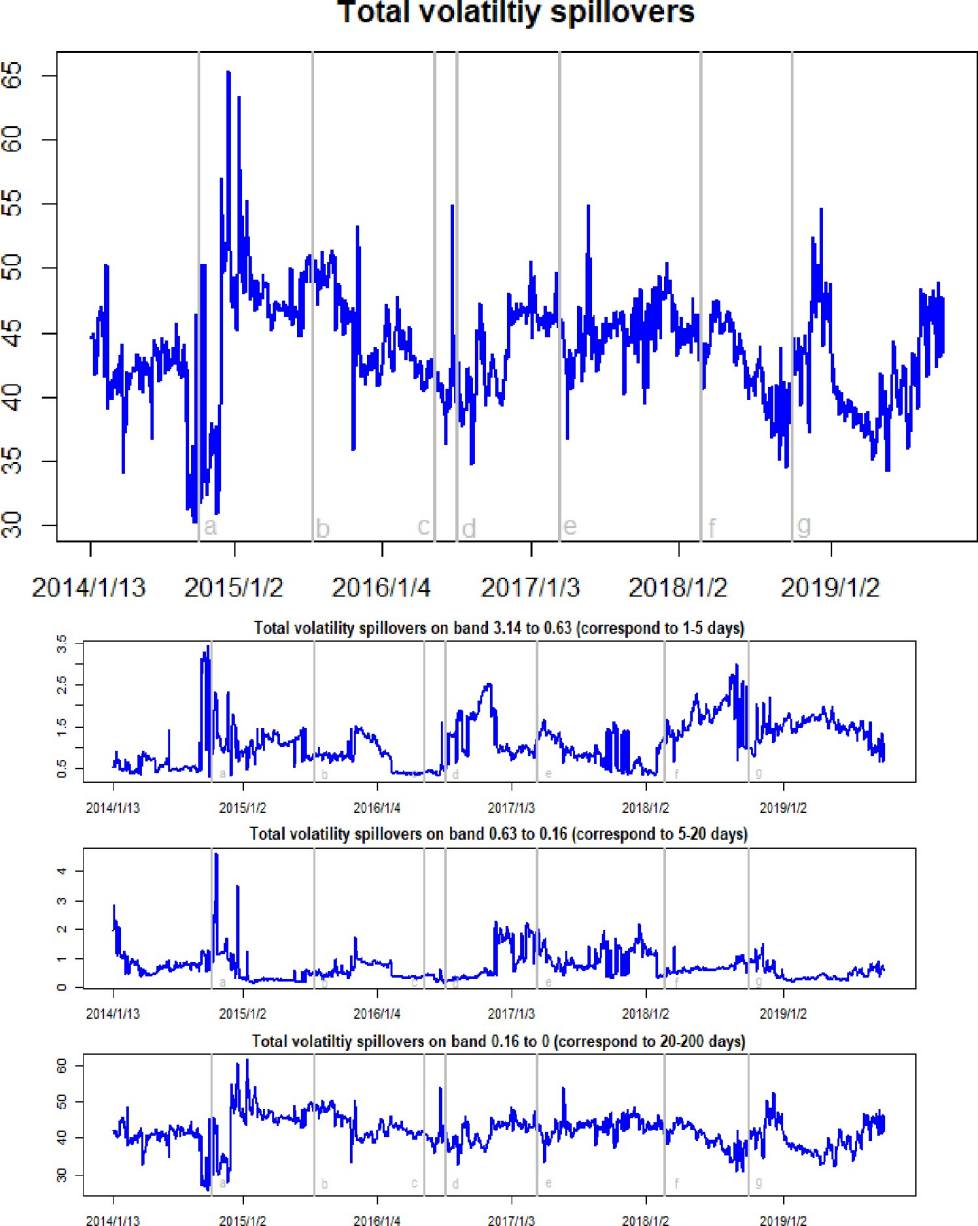
Time-frequency dynamics of total volatility spillovers. a, b, c, d, e, f, g respectively represent the seven events: the WTI oil price collapsed in October 2014, the black swan event in gold market occurred in July 2015, the sharp rise of bitcoin prices in May 2016, the Brexit event in July 2016, the expectations of a Federal Reserve rate hike in March 2017, the price of Bitcoin and gold fluctuating fall at the beginning of 2018, and the WTI oil price declines in August 2018.

Figs 5 and 6 show the time-frequency dynamics of the total return and total volatility spillovers among crude oil, gold, and Bitcoin markets. The salient feature of the total return spillovers is that these are time-varying and manifest cyclical movements over the sample period. The changes in total return spillovers are driven by market events. As the WTI oil price collapsed in October 2014, the total return spillovers present a sharp increase in January 2015. The black swan event in the gold market occurred in July 2015, when the price of gold flash crash down nearly 60 USD in a minute, and Reuters quoted a minimum of 1088.05 USD per ounce, the lowest point in five years. Thus, it can be observed that the total return spillover increases by a jump. Earlier in 2016, with the rebound of the crude oil and gold markets, the total return spillovers remained low. However, because of the sharp rise in Bitcoin prices in May 2016 and the subsequent Brexit event in July, the total return spillover effects here manifest sharp fluctuations. At the beginning of March 2017, expectations of a Federal Reserve rate hike trigged an increase in uncertainties in the crude oil, gold, and Bitcoin markets, meaning that the total return spillovers exhibit sharp jumps. At the beginning of 2018, as the price of Bitcoin and gold fluctuated and fell, the total return spillovers increased in an oscillating manner. Similarly, the declines in crude oil and Bitcoin prices that began at the end of 2018 also led to an increase in total return spillover effects.

In contrast to the total return spillovers, there are two salient features of the total volatility spillovers shown in Fig 6. First, the total volatility spillover effects show a significant increase. The total volatility spillover ranges between 27.36 and 65.56, with an average is 39.17. The average volatility spillover is almost 12 times the average return spillover. This means that, on average, 39.17% of the volatility forecast error variance in all three markets resulted from transmissions. These results are in line with Jin et al. [40], who detected significant volatility spillovers among crude oil, gold, and Bitcoin markets. Second, the total volatility spillover changes significantly following major market events such as the WTI oil price collapse in October 2014, the black swan event in the gold market that occurred in July 2015, the subsequent Brexit event in July 2016, the Federal Reserve rate hike in March 2017, and the crude oil market collapse at the end of 2018.

Taking a closer look at the frequency domain decomposition dynamics of the total return spillovers and total volatility spillovers, we find that the high-frequency components account for an average of 83.8% of total return spillovers. This result is also confirmed in Table 3, in which the high-frequency spillover can be seen to range between 0.47 and 11.3, while the medium-frequency spillover ranges between 0.11 and 1.29, and the low-frequency spillover between 0.03 and 0.86. These data imply that the financial markets process information rapidly, in which a price shock to one asset in the system mainly affects short-term cyclical behavior. However, regarding the total volatility spillovers, as shown in Table 4, it can be seen that the low-frequency component ranges from 25.57 to 61.61, while the high-frequency component ranges from 0.29 and 3.43, and the medium-frequency component from 0.13 to 4.66. The latter indicate that the total volatility spillover effects were primarily created at a low-frequency component. This, in turn, implies that the shocks that create large volatility spillovers in the three markets will impact the system in the long-term. The intuitive thinking behind this is that the asset volatility increases market uncertainty, and that this increasing uncertainty then translates into investors’ more persistent responses to shocks. Thus, the total volatility spillovers translate into long-term uncertainty, and the total volatility spillover effects display high persistence.

**Table 3:** The statistical results of time-frequency dynamics of return spillovers in crude oil, gold, and Bitcoin markets.

	Time domain spillovers			High frequency spillovers			Medium frequency spillovers			Low frequency spillovers		
	Min.	Max.	Mean	Min.	Max.	Mean	Min.	Max.	Mean	Min.	Max.	Mean
Total	0.62	11.71	3.28	0.47	11.30	2.75	0.11	1.29	0.38	0.03	0.86	0.14
Net-oil	-2.11	4.40	-0.05	-2.01	4.52	-0.04	-0.27	0.48	-0.01	-0.12	0.35	-0.001
Net-gold	-3.64	1.27	-0.17	-3.66	1.21	-0.12	-0.47	0.26	-0.03	-0.36	0.12	-0.01
Net-Bitcoin	-1.56	1.90	0.23	-1.85	1.90	0.16	-0.38	0.42	0.04	-0.22	0.19	0.01

**Table 4:** The statistical results of time-frequency dynamics of volatility spillovers in crude oil, gold, and Bitcoin markets.

	Time domain spillovers			High frequency spillovers			Medium frequency spillovers			Low frequency spillovers		
	Min.	Max.	Mean	Min.	Max.	Mean	Min.	Max.	Mean	Min.	Max.	Mean
Total	27.36	65.56	39.17	0.29	3.43	1.11	0.13	4.66	0.71	25.57	61.61	41.88
Net-oil	-28.75	63.87	7.27	-0.28	1.54	0.31	-0.76	3.49	0.17	-27.09	59.93	6.84
Net-gold	-31.82	23.65	-2.61	-1.34	0.43	-0.15	-1.59	1.08	0.03	-30.36	22.73	-0.84
Net-Bitcoin	-32.31	28.33	-4.65	-1.08	0.57	-0.15	-1.99	0.43	-0.20	-29.57	27.57	-6.01

**Fig 7:**
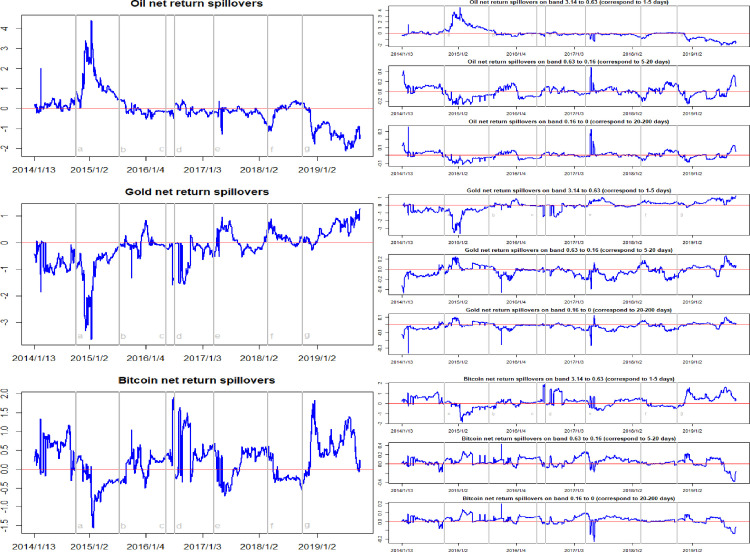
Time-frequency dynamics of net return spillovers for oil, gold, and Bitcoin. a, b, c, d, e, f, g respectively represent the seven events: the WTI oil price collapsed in October 2014, the black swan event in gold market occurred in July 2015, the sharp rise of bitcoin prices in May 2016, the Brexit event in July 2016, the expectations of a Federal Reserve rate hike in March 2017, the price of Bitcoin and gold fluctuating fall at the beginning of 2018, and the WTI oil price declines in August 2018.

**Fig 8:**
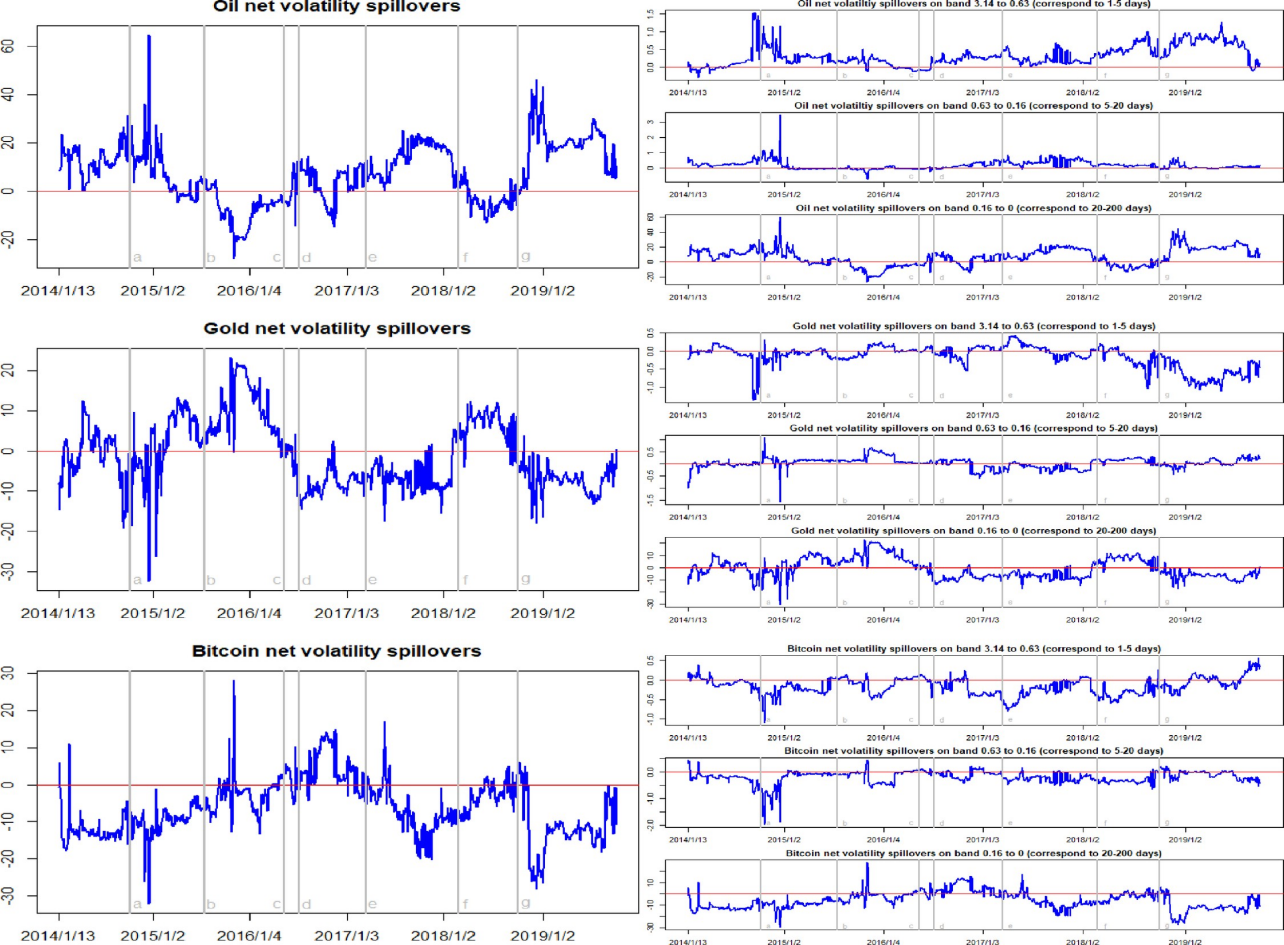
Time-frequency dynamics of net volatility spillovers for oil, gold, and Bitcoin. a, b, c, d, e, f, g respectively represent the seven events: the WTI oil price collapsed in October 2014, the black swan event in gold market occurred in July 2015, the sharp rise of bitcoin prices in May 2016, the Brexit event in July 2016, the expectations of a Federal Reserve rate hike in March 2017, the price of Bitcoin and gold fluctuating fall at the beginning of 2018, and the WTI oil price declines in August 2018.

Figs 7 and 8 report the time-frequency dynamics of the net return spillovers and the net volatility spillovers among crude oil, gold, and Bitcoin markets. Here, we find that the whether an asset market is a receiver or transmitter of return spillovers is differs at different times. Before 2016, the net return spillover effect of crude oil was almost positive. This indicates that the crude oil market is a net transmitter of return spillovers. In particular, when the WTI oil price collapsed in October 2014, the net return spillover effects of crude oil reached the maximum value. However, after 2016, the net return spillover effect of the crude oil market can be seen to be negative most of the time, indicating that the crude oil market is, for the most part, a net receiver of return spillovers. With regard to the gold market, our analysis shows that this was a net receiver of return spillovers in the system for most of time before 2017. However, after 2017, the gold market most consistently became a net transmitter of return spillovers in the system. At the same time, we observe that the net return spillovers of the Bitcoin market lies above zero for a large part of the sample period. This indicates that the Bitcoin market was transmitting return shocks to the crude oil and gold markets for the majority of the sample period. On average, as shown in Table 3, the average net return spillover of the Bitcoin market is 0.23, indicating that this market is the net transmitter of return spillovers in the system. However, the crude oil market and gold market average net return spillovers are -0.05 and -0.17, respectively. This means that, on average, both of these markets are receivers of return spillovers in the system.

Focusing on the net volatility spillovers, we can confirm that the volatility spillovers are bidirectional and asymmetric across the crude oil, gold, and Bitcoin markets. Following major financial events, the net spillover jumps in both negative and positive directions. The crude oil net volatility spillover ranges from -28.75 to 63.87, and is positive for a large part of the sample period. The average net volatility spillover of crude oil is 7.27, as shown in Table 4, implying that the crude oil market, on average, is the transmitter of net volatility spillover effects. Regarding the gold and Bitcoin markets, their net volatility spillovers lie below zero most of the time and their average net volatility spillover effects are negative. This means that the gold and Bitcoin markets, on average, are receivers of net volatility spillovers.

### Robustness checks

**Fig 9:**
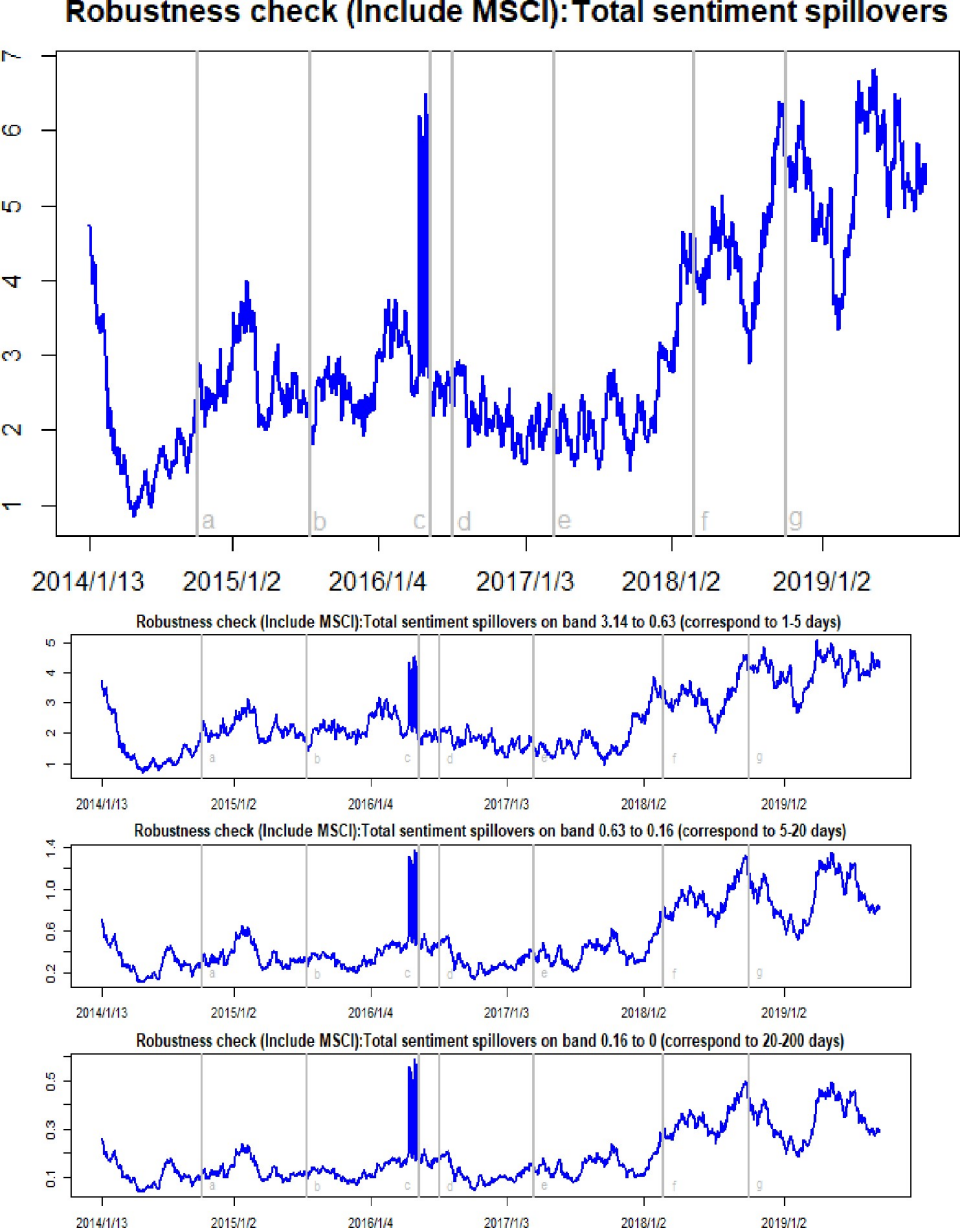
Robustness check (Include MSCI): Time-frequency dynamics of total sentiment spillovers. a, b, c, d, e, f, g respectively represent the seven events: the WTI oil price collapsed in October 2014, the black swan event in gold market occurred in July 2015, the sharp rise of bitcoin prices in May 2016, the Brexit event in July 2016, the expectations of a Federal Reserve rate hike in March 2017, the price of Bitcoin and gold fluctuating fall at the beginning of 2018, and the WTI oil price declines in August 2018.

**Fig 10:**
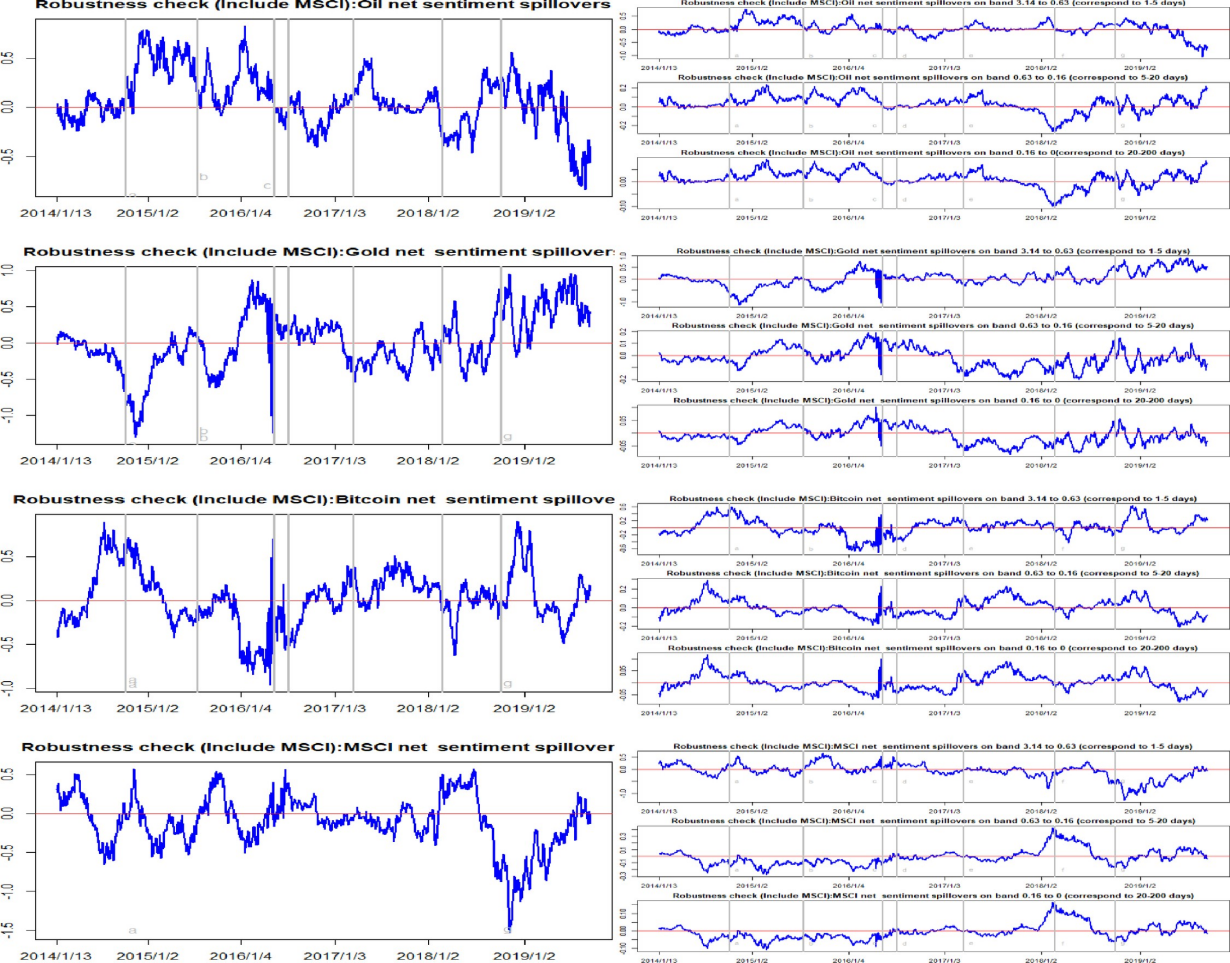
Robustness check (Include MSCI): Time-frequency dynamics of net sentiment spillovers for oil, gold, Bitcoin and MSCI. a, b, c, d, e, f, g respectively represent the seven events: the WTI oil price collapsed in October 2014, the black swan event in gold market occurred in July 2015, the sharp rise of bitcoin prices in May 2016, the Brexit event in July 2016, the expectations of a Federal Reserve rate hike in March 2017, the price of Bitcoin and gold fluctuating fall at the beginning of 2018, and the WTI oil price declines in August 2018.

**Fig 11:**
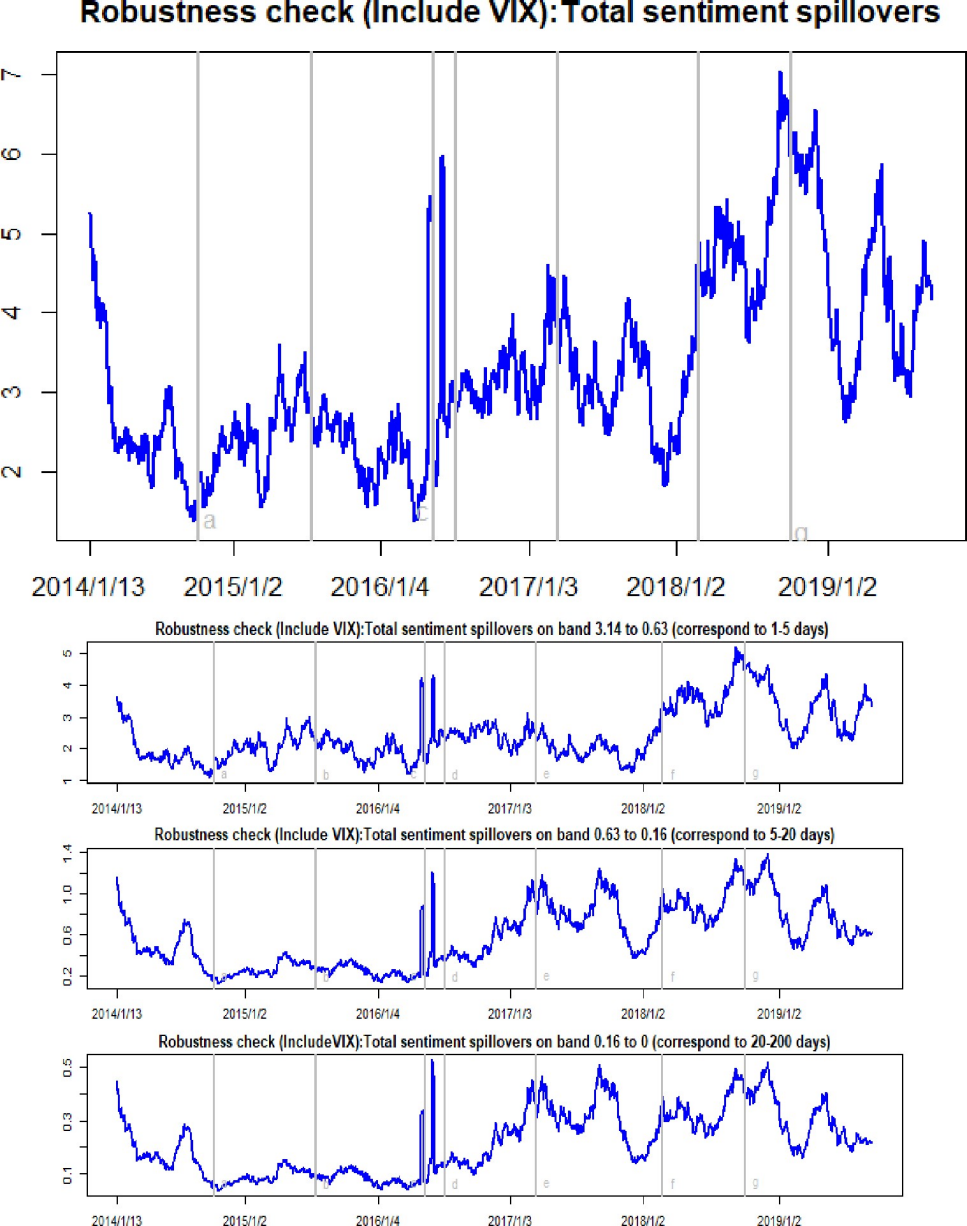
Robustness check (Include VIX): Time-frequency dynamics of total sentiment spillovers. a, b, c, d, e, f, g respectively represent the seven events: the WTI oil price collapsed in October 2014, the black swan event in gold market occurred in July 2015, the sharp rise of Bitcoin prices in May 2016, the Brexit event in July 2016, the expectations of a Federal Reserve rate hike in March 2017, the price of Bitcoin and gold fluctuating fall at the beginning of 2018, and the WTI oil price declines in August 2018.

**Fig 12:**
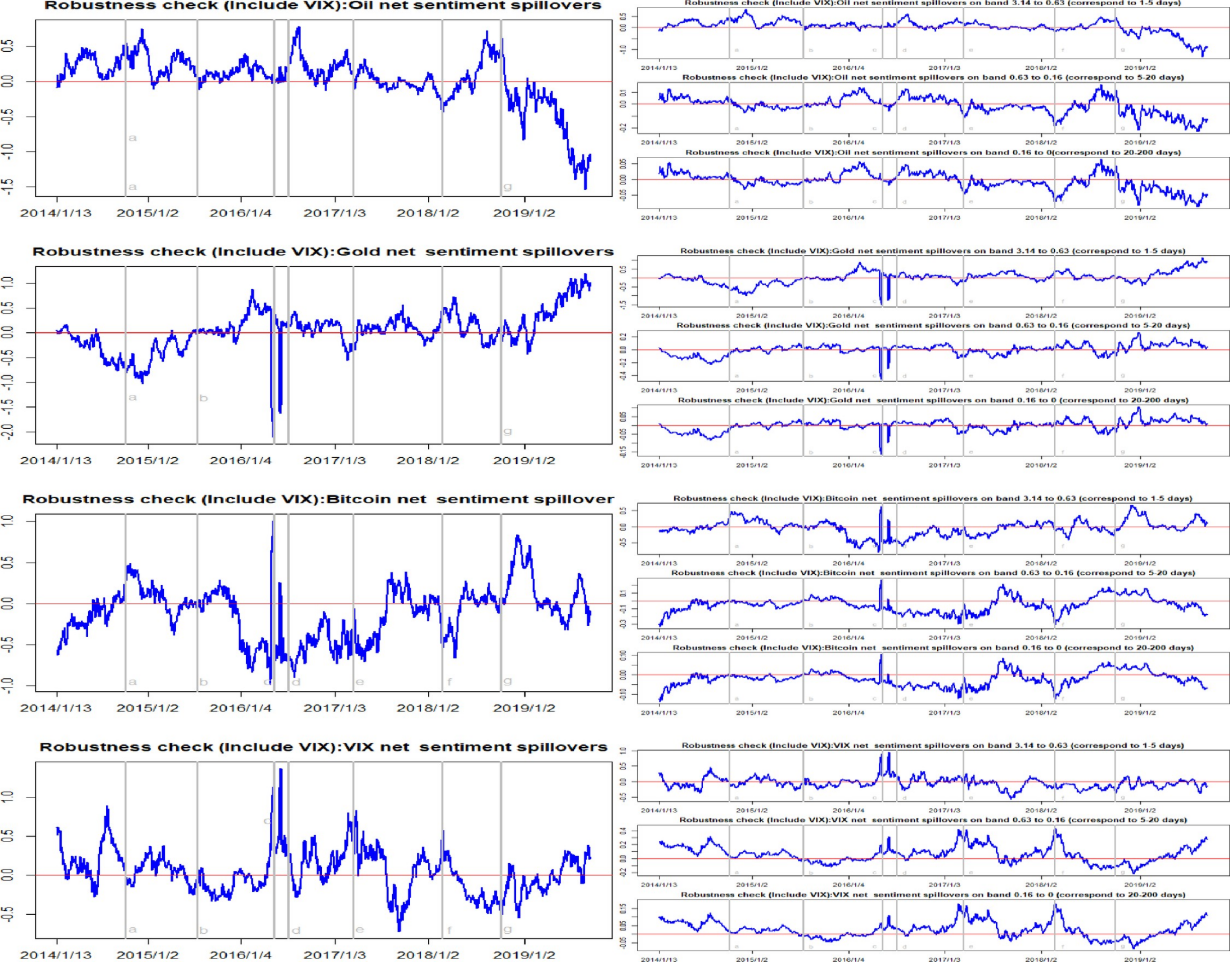
Robustness check (Include VIX): Time-frequency dynamics of net sentiment spillovers for oil, gold, Bitcoin and VIX. a, b, c, d, e, f, g respectively represent the seven events: the WTI oil price collapsed in October 2014, the black swan event in gold market occurred in July 2015, the sharp rise of Bitcoin prices in May 2016, the Brexit event in July 2016, the expectations of a Federal Reserve rate hike in March 2017, the price of Bitcoin and gold fluctuating fall at the beginning of 2018, and the WTI oil price declines in August 2018.

In order to assess the validity of the current results, we conducted several robustness checks. In an earlier study, we only included oil, gold and Bitcoin variables in the spillover matrix. It is well-known that adding a common factor into a VAR model will highly affect both the directionality and the magnitude of spillover effects. We selected the MSCI (Morgan Stanley Capital International) index and the VIX index (implied volatility index) as the common factors, respectively. Figs 9 and 10 summarize the total and net sentiment spillover effects after adding in the MSCI index. Alongside this, Figs 11 and 12 present the total and net sentiment spillover effects across the four variables system included the VIX index. Compared to the earlier results, although the magnitude of the total and net sentiment spillover effects shown in Figs 9–12 manifest subtle changes, the results of the robustness checks are almost consistent with the previous conclusions. The earlier results appear largely robust to other common factors. Overall, the results from these various robustness checks largely support the robustness of the previous findings.

## Conclusions

In this study, we examine the sentiment spillover dynamics among crude oil, gold and Bitcoin markets during the period from April 1, 2013, to September 30, 2019. Specifically, we employed a spectral representation of variance decompositions to decompose the sentiment spillover measurements into short-, medium-, and long-term components. This enabled us to further understand the sentiment spillover dynamics at various frequency bunds. At the same time, in order to compare the differences between sentiment spillover and return and volatility spillovers, we also employed a time-frequency framework to examine the return and volatility spillover effects among the three markets.

We found that the sentiment spillover among the crude oil, gold and Bitcoin markets is time-varying and greatly affected by major market events. These events, such as the WTI oil price collapse in October 2014, the black swan in the gold market that occurred in July 2015, the subsequent Brexit event in July 2016, the Federal Reserve rate hike in March 2017, and the crude oil market collapse at the end of 2018, caused a sharp increase in the sentiment spillover effects. Among the three markets, on average, the Bitcoin market was found to be the major transmitter of sentiment spillover effects, whereas the crude oil and gold markets are the major receivers. Before January 2019, the pairwise sentiment spillover effects between crude oil and gold markets, and crude oil and Bitcoin markets, were negative, implying that the crude oil market is the receiver of sentiment spillovers from the gold and Bitcoin markets. However, the pairwise sentiment spillover values between the crude oil market and the gold and Bitcoin markets are almost positive. Moreover, we found that the high-frequency components of the sentiment spillovers to be significantly greater than the medium- and low-frequency components. This means that the shocks creating sentiment spillovers will impact these markets in the short-term, and that the sentiment shock to one asset in the markets mainly affects short-term cyclical behavior.

We also discovered that the sentiment spillover effects display different characteristics compared with return and volatility spillover effects among the crude oil, gold and Bitcoin markets. We found the following differences: First, the intensity of the volatility spillover emerged as the largest when compared with the return and sentiment spillovers. Second, the Bitcoin market was found to be the major transmitter in the return and sentiments spillover effects, with the gold and Bitcoin markets being the receivers. However, in terms of the volatility spillover effects, the crude oil market was seen to be the major transmitter and the gold and Bitcoin markets the major receivers. Third, the volatility spillover effects were found to be persistent and transmitted for longer periods among the crude oil, gold and Bitcoin markets, whereas the return spillover and sentiment spillover effects occurred rapidly, and had an impact mainly in the short term.

An important implication emerging from this study is that there exist significant time-varying sentiment spillover effects among the crude oil, gold and Bitcoin markets. When transaction demand is driven by investor sentiment, financial assets might be purchased at premium or discount. Therefore, through in-depth understanding of the direction and intensity of sentiment spillovers, short-term investors or speculators can construct a portfolio strategy based on the sentiment spillover index, short selling overvalued assets and buying undervalued assets. Moreover, the results of this study have certain implications for investors and policymakers. By understanding the intensity and frequency components of these spillover effects, investors with different investment horizons can improve their portfolio diversification and hedging strategies when forecasting portfolio market risk exposures among these hedging assets. With regard to policymakers, by closely identifying the major transmitter and receiver of these spillover effects among crude oil, gold and Bitcoin markets over different periods, they can be better prepared to protect against contagion risk and to foster market stability. In the further research, we should focus on the transmission mechanism underlying the discovered spillover dynamics and identify the determinants of such spillover effects.

## Supporting information

S1 File (ZIP)
